# Family functioning and therapeutic success in detoxification in dual diagnosis patients

**DOI:** 10.1192/j.eurpsy.2021.1489

**Published:** 2021-08-13

**Authors:** S. Pombo Chorto

**Affiliations:** Medicine And Surgery, Rovira i Virgili University, Reus, Spain

**Keywords:** dual diagnosis, family functioning, patient dropout, drug detoxification

## Abstract

**Introduction:**

Voluntary dropouts before completion of inpatient psychiatric treatment are common in patients with dual disorders. However, the socioeconomic profile and family structure of patients who undergo a therapeutic program may correlate with their motivation and participation in the treatment received, as well as with their subsequent recovery.

**Objectives:**

To evaluate the family typology of patients with dual diagnosis according to the degree of intrafamily cohesion and adaptability correlated with the prediction of therapeutic success and the risk of voluntary abandonment of hospital detoxification treatment.

**Methods:**

A total of 211 patients admitted to an inpatient psychiatric unit with substance use disorders were studied. Data were obtained from two sources: (1) interview of participants, (2) review of participants’ medical records using the Maudsley Addiction Profile (MAP) and the Family Cohesion and Adaptability Assessment Scale (FACES III).

**Results:**

The 127 subjects who completed the hospital detoxification program had significantly lower MAP and FACES III scores at baseline than the 84 subjects who did not complete the study. Those who did not complete the admission reported greater addictive severity and poorer family functioning. Family cohesion and adaptability measured with FACES III and addictive severity assessed with MAP positively correlated with successful compliance with the hospital treatment program for dual diagnosis patients.

**Conclusions:**

These findings reveal the association of psychosocial and family determinants and addictive severity with treatment completion and subsequent prognostic evolution. Recognizing these predictive characteristics may allow early identification of patients at higher risk of early dropout and prevent it by increasing the intensity of treatment.

